# Presence of zoonotic *Cryptosporidium scrofarum*, *Giardia duodenalis* assemblage A and *Enterocytozoon bieneusi* genotypes in captive Eurasian wild boars (*Sus scrofa*) in China: potential for zoonotic transmission

**DOI:** 10.1186/s13071-016-1942-2

**Published:** 2017-01-06

**Authors:** Wei Li, Lei Deng, Kongju Wu, Xiangming Huang, Yuan Song, Huaiyi Su, Yanchun Hu, Hualin Fu, Zhijun Zhong, Guangneng Peng

**Affiliations:** 1 The Key Laboratory of Animal Disease and Human Health of Sichuan Province, College of Veterinary Medicine, Sichuan Agricultural University, Sichuan, 611130 China; 2 Chengdu Research Base of Giant Panda Breeding, Sichuan, 610081 China

**Keywords:** *Cryptosporidium* spp., *Giardia duodenalis*, *Enterocytozoon bieneusi*, Zoonotic pathogens, Eurasian wild boar, China

## Abstract

**Background:**

*Cryptosporidium* spp*.*, *Giardia duodenalis* and *Enterocytozoon bieneusi* are the main causal pathogens of gastrointestinal disease. However, there are limited reports about the prevalence of these organisms in captive Eurasian wild boars worldwide. Therefore, we examined the occurrence and identified the species/assemblages/genotypes of these pathogens in captive Eurasian wild boars, and estimated the zoonotic potential.

**Findings:**

Of 357 fecal samples collected from captive Eurasian wild boars in China, 155 (43.4%) were infected with *Cryptosporidium*, *G. duodenalis* and/or *E. bieneusi*. The infection rates significantly differed in different areas, but did not differ between wild boars kept indoors and outdoors. Three (0.8%), 11 (3.1%) and 147 (41.2%) fecal samples were positive for *Cryptosporidium*, *G. duodenalis* and *E. bieneusi*, respectively. Sequence analysis of SSU rRNA gene revealed that all of the *Cryptosporidium* strains belonged to *C. scrofarum*. Based on the sequence analysis of the β-giardia gene of *G. duodenalis*, assemblages E and A were characterized. Fourteen *E. bieneusi* genotypes comprising five novel (WildBoar 7–11) and eight known (EbpC, F, CHG19, CHC5, PigEBITS5, D, RWSH4, SC02) genotypes were identified by ITS sequencing. EbpC was the most frequent genotype, detected in 85 specimens. Phylogenetic analysis revealed that all 14 genotypes belonged to Group 1.

**Conclusions:**

This first report on the occurrence of *Cryptosporidium*, *G. duodenalis* and *E. bieneusi* in captive wild boars in China indicates that the presence of zoonotic species/assemblages/genotypes poses a threat to public health. The findings suggest that wild boars could be a significant source of human infection and water pollution.

## Background

Cryptosporidiosis, giardiasis and microsporidiosis are emerging infectious diseases that are mainly caused by the pathogens *Cryptosporidium* spp., *Giardia duodenalis* and *Enterocytozoon bieneusi*, respectively [1]. Humans and animals infected with these protozoan parasites show acute or chronic diarrhea or other symptoms [2]. Currently, over 30 *Cryptosporidium* species are considered valid, and most of the *Cryptosporidium* species, genotypes, or subtypes are host-adapted [3, 4]. *Cryptosporidium suis* and *C. scrofarum*, previously known as pig genotype I and pig genotype II, are the dominant species infecting pigs [5], although *C. muris*, *C. tyzzeri*, *C. parvum*, *C. felis*, *C. hominis*, *C. andersoni* and *C. meleagridis* have also been observed in swine [6]. Given that all of these species were frequently or occasionally found in human infections and have zoonotic potential, they represent a significant public health risk [3, 4]. *Giardia duodenalis*, also known as *G. intestinali*s, or *G. lamblia* is comprised of at least 8 assemblages (assemblages A-H). Assemblages A and B have broad host specificities, having been found in humans and various mammals, while assemblages C-H have strong host specificities and narrow host ranges and assemblages A-F have been identified in pigs [7–9]. *Enterocytozoon bieneusi* is the most common microsporidian species infecting humans. To date, over 200 genotypes have been identified and have been divided into eight groups (Group 1–8) based on phylogenetic analysis; most of the genotypes belonging to Group 1 have zoonotic potential [10]. At least 60 *E. bieneusi* genotypes have been characterized in swine to date [11]. *Cryptosporidium* spp*.*, *G. duodenalis* and *E. bieneusi* are considered to be primarily food-borne and water-borne parasites, posing an invisible threat to public health [12].

Eurasian wild boars (*Sus scrofa*) and domestic pigs (*Sus scrofa domesticus*) belong to the same species (*S. scrofa*), suggesting that they could share the same pathogens, with high potential for transmission between them [13]. Although the presence of *Cryptosporidium* spp*.* and *E. bieneusi* has been reported in domestic pigs, no survey on the occurrence of *G. duodenalis* in swine of China has been conducted. Domestic pigs in Chongqing, Shaanxi, Shanghai, Heilongjiang, Henan, Jiangsu and Taiwan were found to be infected with *Cryptosporidium* spp., with the infection rates ranging from 3.3 to 55.8%, and *C. suis*, *C. scrofarum*, *C. andersoni* and *C. tyzzeri* were the species identified [14–20]. Several reports have revealed the existence of *E. bieneusi* in domestic pigs in China, and over 20 genotypes have been identified, including CS-1, CS-3, CS-4, CS-6, CS-9, CS-10, EbpA, EbpB, EbpC, EbpD, Henan-I, Henan-IV, G, D, H, O, LW1, CHN1, CHN7, CHN8, CHN9, CHN10, EBITS3, PigEBITS5 and HLJ-I to HLJ-IV, with most of the identified genotypes confirmed to be zoonotic [21–25].

Wild boars have a worldwide distribution. They not only provide meat for human beings but are also widely used in scientific research. Unfortunately, wild boars are readily exposed and susceptible to parasites such as helminths and/or protozoa. To date, there is no published report on the occurrence of *Cryptosporidium* spp., *G. duodenalis* and *E. bieneusi* in wild boars in China. Therefore, the aim of this study was to identify the species/assemblages/genotypes using molecular characterization. Moreover, the role of wild boars as a potential reservoir of protozoa for other animals and human beings was estimated.

## Methods

### Sample collection

During 2014 to 2015, a total of 357 fecal samples were collected from captive wild boars in four sites of Sichuan province, including 239, 60, 50 and 8 specimens collected from Aba, Mingshan, Qionglai and Hanyuan, respectively. Among them, 308 and 49 were from wild boars kept indoors and outdoors, respectively. During specimen collection, we only gathered the top layer of the feces to ensure no contamination of the samples. The specimens were stored in centrifuge tubes containing 2.5% potassium dichromate and then placed in containers filled with ice packs and transported to the laboratory immediately.

### DNA extraction and nested PCR amplification

Before extracting DNA, the fecal samples were washed with distilled water until the potassium dichromate was removed. Subsequently, genomic DNA was extracted from approximately 200 mg of semi-purified product using the E.Z.N.A Tool DNA Kit (D4015–02; Omega Bio-Tek Inc., Norcross, GA, USA) following the manufacturer’s instructions. DNA samples were stored in 200 μl of the kit’s Solution Buffer at -20 °C until use.

*Cryptosporidium* spp*.*, *G. duodenalis* and *E. bieneusi* were identified by nested PCR amplification of the small subunit (SSU) rRNA gene, β-giardin (bg), and internal transcribed spacer (ITS) genes, respectively. The primers and annealing temperatures were previously reported [26]. For *Cryptosporidium* spp*.* and *E. bieneusi*, the annealing temperature was 55 °C for both primary and secondary PCR amplification. For *G. duodenalis* detection, the annealing temperatures of 65 °C and 55 °C were used in the primary and secondary PCR amplification, respectively. The secondary PCR products were visualized by staining with Golden View following 1% agarose gel electrophoresis.

### Data analysis

The amplicons of the expected size were sent to Invitrogen (Shanghai, China) for sequencing. To ensure sequence accuracy, two-directional sequencing methods were used. To determine *Cryptosporidium* species, *G. duodenalis* assemblages and *E. bieneusi* genotypes, the sequences obtained in this study were aligned with sequences downloaded from the GenBank database *via* BLAST analysis (http://blast.ncbi.nlm.nih.gov) and using ClustalX software. For *E. bieneusi*, phylogenetic analysis of ITS sequences was performed using Mega software [27], and neighbor-joining phylogenetic analysis of the aligned *E. bieneusi* sequences was utilized to assess genetic clustering of genotypes. A total of 1,000 replicates were used for bootstrap analysis.

The infection rates between animals in different areas and with different farming modes (indoor or outdoor) were compared using the Chi-square test. A *P*-value < 0.05 was considered to indicate a significant difference. All tests were conducted using SPSS version 17.0 software (SPSS Inc., Chicago, IL, USA).

### Nucleotide sequence GenBank accession numbers

All of the nucleotide sequences of SSU rRNA and the β-giardin gene from wild boars obtained in this study were deposited in the GenBank database under the accession numbers KU668893, KU668897 and KU668898 for *Cryptosporidium*, and KU668880, KU668883–KU668892 for *G. duodenalis*. The representative *E. bieneusi* sequences were deposited in the GenBank database under the accession numbers KX670577–KX670590.

## Results and discussion

In our study, mixed infection was detected in six specimens, and all cases involved the combination of *G. duodenalis* and *E. bieneusi*. Considering all three pathogens evaluated in this study, a total of 155 individuals were infected, for an overall infection rate of 43.4%. Studies on these three pathogens in China have thus far focused on wildlife in captivity, companion animals, domestic animals and wastewater [26, 28, 29]. Here, we provide the first report of the prevalence and genetic characteristics of *Cryptosporidium*, *G. duodenalis* and *E. bieneusi* in captive wild boars in China, with infection rates of 0.8%, 3.1% and 41.2%, respectively, suggesting that the presence of *E. bieneusi* is generally more common. Considering the pathogens together, the infection rates were 35.6% (85/239), 60% (36/60), 66% (33/50) and 12.5% (1/8) in Aba, Mingshan, Qionglai and Hanyuan, respectively. A significant difference was observed between different areas (*χ* ^2^ = 26.207, *df* = 3, *P* = 0.001), which confirms the results of a study conducted on *E. bieneusi* infection in farmed foxes in northern China [30]. However, in the previous study, the prevalence was found to be highly associated with the farming mode, whereas in the present study, the infection rates were not significantly different (*χ* ^2^ = 0.156, *df* = 1, *P* > 0.05) between the wild boars kept indoors (135/308; 43.8%) and those kept outdoors (20/49; 40.8%) [30].

For *Cryptosporidium* spp., three *Cryptosporidium*-positive fecal samples were detected in Qionglai (*n* = 2) and Hanyuan (*n* = 1), and identified as *C. scrofarum* (Tables 1, 2). To date, infection of *Cryptosporidium* in wild boars has mainly been reported in European countries, with a prevalence of 7.6–16.7% in Spain, 16.5–16.9% in the Czech Republic, 18.2% in Austria, 0–8.5% in Poland and 5.4% in the Slovak Republic [6, 31–34]. Furthermore, *C. scrofarum*, *C. suis*, *C. parvum*, and mixed infection of *C. scrofarum* with *C. suis* have been identified in Europe, and *C. scrofarum* was the predominant *Cryptosporidium* species, followed by *C. suis* (Table 3). No other *Cryptosporidium* species that can infect domestic pigs has been detected in wild boars thus far. In our study, only three wild boars (0.8%) were found to be infected with *Cryptosporidium*, suggesting a much lower infection rate compared to those reported elsewhere, and only *C. scrofarum* was identified. Collectively, these findings suggest that wild boars might mainly harbor *C. scrofarum* and *C. suis*, which are both porcine-specific species, having been reported in domestic pigs worldwide [33]. *Cryptosporidium scrofarum* is recognized as a zoonotic species, and has been detected in humans, domestic animals, and even source water [20, 35, 36]. Thus, wild boars can serve as an environmental reservoir of *Cryptosporidium* transmitted to animals, humans and water.

**Table 1:** Occurrence of *Cryptosporidium* spp., *G. duodenalis* and *E. bieneusi* in captive Eurasian wild boars in this study

Factor	Category	No. of tested	No. of positive (%) [95% CI]		
			*Cryptosporidium* spp*.*	*G. duodenalis*	*E. bieneusi*
Area	Aba	239	0 (0)	0 (0)	85 (35.6) [0.295–0.417]
	Mingshan	60	0 (0)	1 (1.67) [-0.017–0.050]	36 (60.0) [0.472–0.728]
	Qionglai	50	2 (4.0) [-0.016–0.096]	10 (20.0) [0.085–0.315]	26 (52.0) [0.377–0.663]
	Hanyuan	8	1 (12.5) [-0.171–0.421]	0 (0)	0 (0)
	Subtotal	357	3 (0.8) [-0.001–0.018]	11 (3.1) [0.013–0.049]	147 (41.2) [0.361–0.463]
Mode	Indoor	308	3 (1.0) [-0.001–0.021]	11 (3.6) [0.015–0.057]	127 (41.2) [0.357–0.468]
	Outdoor	49	0 (0)	0 (0)	20 (40.8) [0.266–0.551]
	Subtotal	357	3 (0.8) [-0.001–0.018]	11(3.1) [0.013–0.049]	147 (41.2) [0.361–0.463]

**Table 2:** Distribution of *Cryptosporidium* species, *G. duodenalis* assemblages and *E. bieneusi* genotypes in captive Eurasian wild boars in this study

Area	*Cryptosporidium* spp.	*G. duodenalis* assemblages	*E. bieneusi* genotypes
Aba			EbpC (69); CHG19 (8); CHC5 (5); F (2); SC02 (1)
Mingshan		A (1)	F (20); WildBoar 10 (6); WildBoar 8 (5); WildBoar 9 (2); WildBoar 7 (1); EbpC (1); PigEBITS5 (1)
Qionglai	*C. scrofarum* (2)	A (1); E (9)	EbpC (15); CHC5 (5); CHG19 (3); D (1); WildBoar 11 (1); RWSH4 (1)
Hanyuan	*C. scrofarum* (1)		
Total	*C. scrofarum* (3)	A (2); E (9)	EbpC (85); F (22); CHG19 (11); CHC5 (10); WildBoar 10 (6); WildBoar 8 (5); WildBoar 9 (2); WildBoar 7 (1); PigEBITS5 (1); D (1); WildBoar 11 (1); RWSH4 (1); SC02 (1)

**Table 3:** Occurrence of *Cryptosporidium* spp. and *G. duodenalis* in swine reported worldwide

Pathogen	Host	Country	Infection rate (%) (No. infected/No. examined)	Species/assemblage	Reference
*Cryptosporidium*	Wild boar	Spain	16.7 (35/209)	*C. scrofarum* (19), *C. suis* (5), *C. parvum* (3)	[6]
	Wild boar	Spain	7.6 (29/381)		[31]
	Wild boar	Czech Republic	16.5 (32/193)	*C. scrofarum* (7), *C. suis* (13), *C. scrofarum* + *C. suis* (12)	[32]
	Wild boar	Czech Republic	16.9 (39/231)	*C. scrofarum* (14), *C. suis* (13), *C. scrofarum* + *C. suis* (12)	[33]
	Wild boar	Austria	18.2 (8/44)	*C. scrofarum* (3), *C. suis* (2), *C. scrofarum* + *C. suis* (3)	[33]
	Wild boar	Poland	8.5 (11/129)	*C. scrofarum* (8), *C. suis* (1), *C. scrofarum* + *C. suis* (2)	[33]
	Wild boar	Poland	0 (0/5)		[34]
	Wild boar	Slovak Republic	5.4 (3/56)	*C. scrofarum* (1), *C. suis* (2)	[33]
Subtotal			12.6 (157/1248)	*C. scrofarum* (52), *C. suis* (36), *C. parvum* (3), *C. scrofarum* + *C. suis* (29)	
*G. duodenalis*	Wild boar	Spain	1.3 (5/381)		[31]
	Wild boar	Poland	0 (0/5)		[34]
	Wild boar	Croatia	1.4 (2/144)	A (1)	[37]
Subtotal			1.3 (7/530)	A (1)	
*G. duodenalis*	Domestic pig	Austrila	31.1 (90/289)	A (19), E (37), F (1)	[38]
	Domestic pig	Brazil	3.4 (3/90)	E (2), D (1)	[39]
	Domestic pig	Denmark	17.4 (215/1237)		[40]
	Domestic pig	Canada	66.4 (81/122)	B (58), E (5)	[9]
	Domestic pig	Canada	1.0 (6/633)		[41]
	Domestic pig	UK	57.1 (4/7)	C (1), F (2)	[8]
	Domestic pig	Norway	1.5 (10/684)		[42]
Subtotal			13.4 (409/3062)	B (58), E (44), A (19), C (1), D (1), F (3)	

We found a prevalence of 3.1% for *G. duodenalis* (Table 1), which is higher than that detected in wild boars in Poland (0%), Croatia (1.4%), and Spain (1.3%) [31, 34, 37]. In contrast, the prevalence in domestic pigs has been reported to vary from 1 to 66.4% (Table 3) [38–42]. Reports on *G. duodenalis* infection in wild boars are limited, and only one wild boar isolate was successfully identified using a molecular method, which turned out to be a part of assemblage A. In our sample, 11 wild boars were found to be infected with *G. duodenalis*. Specifically, two were infected with assemblage A and nine were infected with assemblage E (Table 2). Based on epidemiological data, assemblages A-F have been found in domestic pigs [7]. Among them, assemblages A and E were both found in domestic pigs and wild boars, and the latter was the most prevalent genotype, suggesting the possibility of inter-species transmission; however, the species of origin is not clear at present. A recent study conducted in Rio de Janeiro, Brazil detected that 15 people were infected with assemblage E, which further demonstrated that humans can be infected with assemblage E through an anthropozoonotic cycle [43]. Thus, the dominant assemblage E, and assemblage A detected in our study could be transmitted to humans through an anthropozoonotic transmission cycle.

In the present study, a total of 147 specimens were found to be *E. bieneusi*-positive, forming 14 *E. bieneusi* genotypes comprising five novel genotypes (WildBoar 7–11) and eight known genotypes (EbpC, F, CHG19, CHC5, PigEBITS5, D, RWSH4 and SC02), as identified by ITS sequencing analysis (Tables 1, 2). The most frequent genotype was EbpC, followed by genotype F (Table 2). The first report of wild boar *E. bieneusi* infection was in Poland, but this was confirmed by chromotrope 2R and fluorescence *in situ* hybridization analyses, and the genotype was not characterized [44]. We could only found one published paper reporting the prevalence and genetic characterization of *E. bieneusi* in wild boars: 11 genotypes were found in Central Europe (D, EbpA, EbpC, G, Henan-I, and WildBoar1–6), and EbpA was the most frequently detected microsporidian, which is different from our results [13]. Furthermore, the infection rate of the previous study was only 7.17% (33/460), which is markedly lower than that detected in our study (41.2%) [13]. These differences between the two studies may be due to the different geographic areas and living conditions. The novel genotypes (WildBoar 1–11) identified in wild boars of China may indicate that *E. bieneusi* in wild boars has relatively higher genetic variability. The genotypes EbpC, D, and PigEBITS5 previously identified in domestic pigs were also detected in wild boars. However, ten genotypes (F, CHG19, CHC5, RWSH4, SC02, WildBoar 7–11) have only been detected in wild boars to date. Thus, further studies in swine are needed to determine whether or not these ten genotypes have the capacity to infect domestic pigs, and if the genotypes found in domestic pigs can infect wild boars. This is the first report of these ten genotypes in wild boars, which may indicate that the wild boar is a new host for *E. bieneusi* of these genotypes.

**Fig. 1:**
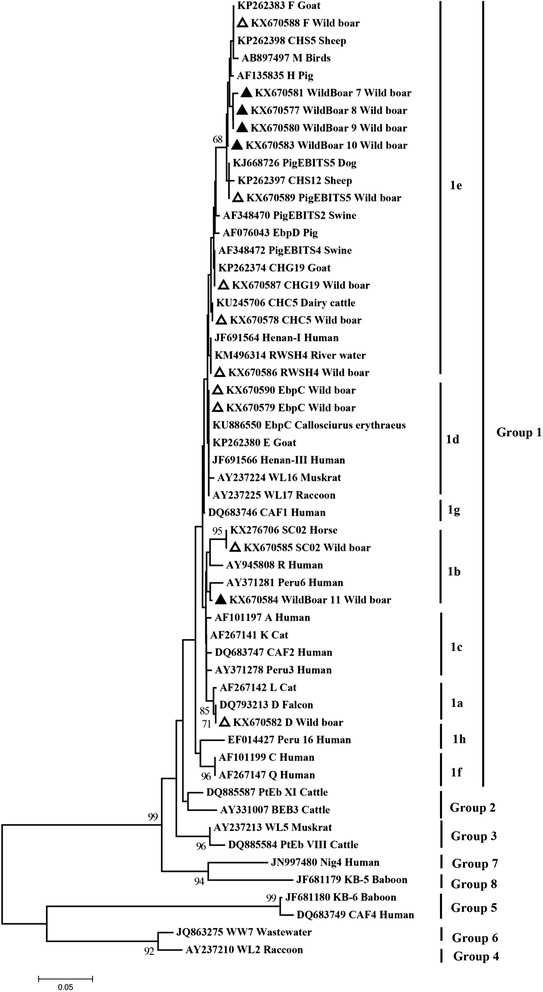
Phylogenetic relationships of ITS nucleotide sequences of the *Enterocytozoon bieneusi* genotypes identified in this study and other reported genotypes. The phylogeny was inferred by a neighbor-joining analysis. Bootstrap values were obtained using 1,000 pseudo-replicates and those greater than > 50% are shown on nodes. The genotypes identified in this study are marked by outlined triangles and the novel genotypes are marked by filled triangles

Based on the phylogenetic analysis of the ITS gene, we found that all of the 14 *E. bieneusi* genotypes identified in our study belong to Group 1 (Fig. 1), which suggests their zoonotic potential. Among them, the human-pathogenic genotype EbpC was the most prevalent, which has been frequently found in hospitalized children in Shanghai, HIV+ and HIV- people in Henan, wastewater in four cities, and pigs in multiple cities in China [1, 21, 23–25, 45, 46]. These studies suggest that pigs might be a source of human microsporidiosis and water pollution. Moreover, EbpC may represent the main cause of human microsporidiosis in China [21, 23–25]. Our results confirm this possibility by demonstrating the potential for the zoonotic transmission of *E. bieneusi*. Furthermore, the identification of five novel genotypes has broadened the recognized genotypes and suggests high genetic variability among *E. bieneusi*.

## Conclusions

We provide the first report on the presence of *Cryptosporidium*, *G. duodenalis* and *E. bieneusi* in captive wild boars from China, and identified *C. scrofarum*, assemblages A/E, and 14 *E. bieneusi* genotypes. Most of the species/assemblages/genotypes identified have been detected in humans. Our results revealed that wild boars and domestic pigs can share the same pathogens, and that wild boars could be an important source of animal and human cryptosporidiosis, giardiasis and microsporidiosis, as well as water contamination. Thus, measures should be taken to control the possible transmission. Furthermore, more molecular epidemiological surveys of *Cryptosporidium*, *G. duodenalis* and *E. bieneusi* in wild boars, humans, animals and water samples are needed to better elucidate the transmission risk and mode.

## Abbreviations

Bg: *β*-giardin
ITS: Internal transcribed spacer
SSU rRNA: The small subunit rRNA
